# Strawberry and Drupe Fruit Wines Antioxidant Activity and Protective Effect Against Induced Oxidative Stress in Rat Synaptosomes

**DOI:** 10.3390/antiox14020155

**Published:** 2025-01-28

**Authors:** Uroš Čakar, Mirjana Čolović, Danijela Milenković, Maja Pagnacco, Jelena Maksimović, Danijela Krstić, Brižita Đorđević

**Affiliations:** 1 Department of Bromatology, Faculty of Pharmacy, University of Belgrade, 11 000 Belgrade, Serbia; brizita.djordjevic@pharmacy.bg.ac.rs; 2 Department of Physical Chemistry, “Vinča” Institute of Nuclear Sciences-National Institute of the Republic of Serbia, University of Belgrade, 11 351 Belgrade, Serbia; colovicm@vin.bg.ac.rs; 3 Department of Physics and Mathematics, Faculty of Pharmacy, University of Belgrade, 11 000 Belgrade, Serbia; danijela.milenkovic@pharmacy.bg.ac.rs; 4 Department of Catalysis and Chemical Engineering, Institute of Chemistry, Technology and Metallurgy, University of Belgrade, 11 000 Belgrade, Serbia; maja.pagnacco@nanosys.ihtm.bg.ac.rs; 5 Faculty of Physical Chemistry, University of Belgrade, 11 158 Belgrade, Serbia; jelena.maksimovic@ffh.bg.ac.rs; 6 Institute of Medical Chemistry, Faculty of Medicine, University of Belgrade, 11 000 Belgrade, Serbia; danijela.krstic@med.bg.ac.rs

**Keywords:** Briggs–Rauscher (BR) reaction, catalase (CAT), glutathione peroxidase (GPx), hierarchical cluster analysis (HCA), malondialdehyde (MDA), principal component analysis (PCA), superoxide dismutase (SOD)

## Abstract

The aim of this study was to investigate the antioxidant capacity of fruit wines and their protective effects against hydrogen peroxide-induced oxidative stress in rat synaptosomes in vitro. The wines were produced from strawberries and drupe fruits (i.e., plum, sweet cherry, peach, and apricot) through microvinification with a pure *S. cerevisiae* yeast culture. Fruit wines were produced with and without added sugar before the start of fermentation, whereas subvariants with and without pits were only applied to drupe fruit wines. First, synaptosomes were treated with the wines, while oxidative stress was induced with H_2_O_2_. Subsequently, the activities of antioxidant enzymes (superoxide dismutase (SOD), catalase (CAT), and glutathione peroxidase (GPx)) and the content of malondialdehyde (MDA), an indicator of membrane injury, were determined. In addition, the Briggs–Rauscher reaction (BR) was used to evaluate the inhibition capacity against free radicals. All investigated fruit wines increased the activity of the studied antioxidant enzymes and decreased MDA content compared to the corresponding controls (synaptosomes treated with H_2_O_2_). After synaptosomal treatment with plum wine, the highest activities were observed for SOD (5.57 U/mg protein) and GPx (0.015 U/mg protein). Strawberry wine induced the highest CAT activity (0.047 U/mg protein) and showed the best ability to reduce lipid peroxidation, yielding the lowest MDA level (2.68 nmol/mg). Strawberry, plum, and sweet cherry wines were identified as samples with higher antioxidant activity in both principal component analysis (PCA) and hierarchical cluster analysis (HCA). Finally, plum wine exhibited the highest inhibitory activity in the BR reaction (397 s). The results suggest that fruit wines could be considered potential functional food due to their protective effects against oxidative stress.

## 1. Introduction

The oxidative metabolism of eukaryotic cells takes place in the mitochondria and the endoplasmic reticulum. During this process, free radicals are generated, among which reactive oxygen species (ROS) should be emphasized as metabolic by-products of physiological redox processes [1]. In healthy cells, the rate of this process is tightly regulated. The human body has a system responsible for maintaining oxidative balance, which consists of endogenous cellular antioxidant enzymes such as superoxide dismutase (SOD), catalase (CAT), and glutathione peroxidase (GPx), serving as the first line of defense against free radicals [2]. In addition to antioxidant enzymes, non-enzymatic antioxidants such as polyphenols, vitamins, and other biologically active compounds from fruits, vegetables, and products made from them are involved in this process. The disruption of the enzymatic antioxidant system and the insufficient intake of antioxidants can lead to the excessive formation of ROS, ultimately resulting in the development of oxidative stress [3].

Biomolecules such as proteins, nucleic acids, and cell membrane lipids are susceptible to oxidative stress, leading to changes in their structure and affecting normal organism functions. Alongside physiologically generated ROS, exogenous sources such as xenobiotics, radiation, and other environmental pollutants can contribute to abnormal ROS formation [4]. As highly reactive molecules, ROS are implicated in the development of pathological processes in humans and animals. The imbalance between the formation and scavenging of ROS is responsible for the onset of oxidative stress, impairment of cell function, and tissue inflammation [5]. Inflammatory processes underlie the development of chronic, non-communicable diseases that are prevalent worldwide. Alterations in cell and tissue integrity lead to the development of pathological conditions such as cardiovascular diseases, metabolic syndrome, neurodegenerative disorders, and carcinogenesis [6,7,8,9].

The antioxidant enzyme system is rapidly activated when ROS are produced in excessive amounts. Achieving homeostasis between the generation and scavenging of free radicals is essential to prevent further chain reactions that can lead to the development of pathological processes in the human organism [6]. Increased activity of antioxidant enzymes effectively protects cells from oxidative stress but non-enzymatic antioxidants are also involved in scavenging ROS. In particular, polyphenols, such as phenolic acids and flavonoids, show significant antioxidant capacity against oxidative stress [10,11,12]. Previous studies have reported varying effects of phenolic compounds from natural products on the activity of antioxidant enzymes; activation was shown, while in some cases, inhibition was observed. Plant polyphenols from grape pomace, fruits, and green tea have been shown to increase the activity of antioxidant protection enzymes in different animal models such as rats [13,14] and rabbits [15], whereas another study found decreased activity [16]. In a study conducted on human cells treated with berry fruit extracts rich in phenolic compounds, increased activities of SOD, CAT, and GPx were observed [17].

Various methods can be employed to determine the antioxidant activity of fruits and products made from them. In this work, information on the antioxidant activity of fruit wines was also obtained by the Briggs–Rauscher (BR) oscillation reaction. This reaction can be characterized as a chemical system used to measure the total antioxidant activity of various natural products derived from biologically active compounds in plants [18,19,20]. The determination of antioxidant activity is based on the reactions between the samples and their biologically active compounds with radicals (HO^•^, HOO^•^, I^•^, IO_2_^•^, etc.) that are potentially present in the BR reaction [21]. In contrast to methods that utilize stable artificial radicals (DPPH, ORAC, ABTS) to determine antioxidant activity, the BR reaction generates reactive oxygen and iodine radicals that are present in living organisms. As a result of these reactions, quenching of the oscillatory dynamics occurs, which can be used to assess the antioxidant activity of different samples [22]. It is important to emphasize that the experimental conditions for the BR method in this work are similar to physiological conditions; specifically, the temperature was maintained at about 37 °C, and the pH of the BR reaction solution was approximately 2, corresponding to the pH of gastric acid. This makes the reaction suitable for studying the antioxidant capacity of biological agents, which could indicate their potential activity in the human organism [22].

Recent publications have shown that strawberries, drupe fruits (such as plum, sweet cherry, apricot, and peach), and their wines are a rich source of biologically active compounds with antioxidant properties. For example, anthocyanins have been detected in strawberry and cherry wines [23,24], while studies on various strawberry and sweet cherry varieties revealed the presence of hydroxybenzoic acid derivatives (specifically, protocatechuic acid and vanillic acid) [25,26]. An earlier study on the phenolic profile of plums found chlorogenic acid to be predominant [27], and plum wine was reported to have high phenolic content and significant antioxidant activity [28]. Similarly, apricot and peach wines showed antiradical properties against stable artificial radicals (DPPH and ABTS), while phenolic acids (gallic and chlorogenic acids) and flavonoids (catechin and quercetin) predominated in the fruits and wines [29,30]. In addition to the presence of specific phenolic compounds in these fruits and wines, it is important to point out their significant health-promoting effects [10]. As indicated earlier, the potential of fruit wines to prevent experimentally induced oxidative stress in isolated animal cells has not been investigated in previous studies.

This study aimed to investigate the antioxidant capacity of fruit wines and their protective effects against hydrogen peroxide-induced oxidative stress in rat synaptosomes in vitro. The activity of strawberry and drupe fruit wines (plum, sweet cherry, peach, and apricot) produced through a controlled microvinification procedure was investigated. The antioxidant activity of the fruit wines was estimated in isolated rat synaptosomes by monitoring the activities of antioxidant enzymes (SOD, CAT, and GPx) and malondialdehyde (MDA) levels as an indicator of cell membrane damage. Finally, the BR method was applied to evaluate the antioxidant capacity of the fruit wines and their biologically active compounds at the pH value representative of the human stomach.

## 2. Materials and Methods

### 2.1. Chemicals and Reagents

All chemicals were of analytical grade. Glutathione reductase from baker’s yeast (*Saccharomyces cerevisiae*) (205 units/mg protein, 2.2 mg protein/mL), reduced glutathione (GSH), EDTA, a SOD determination kit, NADH, NADPH, thiobarbituric acid (TBA), and Folin–Ciocalteu reagent were from Sigma-Aldrich (Steinheim, Germany). Other reagents such as sodium dihydrogen phosphate, sodium hydrogen phosphate, sodium carbonate, sucrose, hydrochloric acid, *n*-butanol, malonic acid, manganese sulfate, perchloric acid, and potassium iodate were purchased from Merck (Darmstadt, Germany).

### 2.2. Wine Production

Strawberries, sweet cherries, plums, peaches, and apricots were used to produce wines. Strawberries (*Fragaria x ananassa*) and plums (*Prunus domestica*) were harvested from the orchards around the town of Valjevo in western Serbia. Peaches (*Prunus persica*), apricots (*Prunus armeniaca*), and sweet cherries (*Prunus avium*) were obtained from the orchards in Grocka, a municipality in the Serbian capital Belgrade. All fruits were harvested in 2017. The wines were produced under controlled conditions through experimental microvinifications [31].

### 2.3. Lyophilization of Samples

This procedure was used to prevent the effects of ethanol from wine samples on lipid peroxidation and enzyme activities during the experiment. For this purpose, a laboratory freeze dryer, Christ Alpha 1-2/LD plus (Osterode am Harz, Germany), was used (main drying time: 8.5 h; final drying: 30 min). The operating parameters of the device were a pressure of 0.30 mbar and a temperature of −55 °C for 9 h. Lyophilized wine samples were used for further analysis.

### 2.4. Preparation of Synaptosomes

Three-month-old male *Wistar albino* rats weighing 180–220 g were housed in carbon cages (3–4 per cage) and had ad libitum access to standard rodent pellet food and tap water. Environmental conditions were kept constant: 12 h/12 h dark/light cycle, 23 ± 2 °C, and a humidity of 55 ± 10%. Rats were anesthetized and decapitated using a guillotine (Harvard Apparatus, Holliston, MA, USA) following Directive 2010/63/EU and Good Laboratory Practice of the European Convention for the Protection of Vertebrate Animals used for Experimental and other Scientific Purposes. This study was conducted according to the guidelines of the Declaration of Helsinki and approved by the Ethics Committee for Welfare Protection of Experimental Animals of the Faculty of Medicine, University of Belgrade (protocol code: 323-07-04764/2012-05, 8 October 2012, issued by the Ministry of Agriculture, Forestry, and Water Protection, Veterinary Directorate). Synaptosomes were isolated according to the reported procedure [32]. In brief, whole brains were taken out and homogenized in 10 mL of cold 0.32 mol/L sucrose solution buffered with 10 mmol/L Tris–HCl, pH 7.4. The homogenates were centrifuged at 1000× *g* (YGL10MA refrigerated centrifuge, Yonglekang, Changsha, China) at 4 °C for 10 min. The separated supernatants were collected and the precipitated fractions were re-suspended and processed as described above. After the collected supernatants were centrifuged at 10,000× *g* (YGL10MA refrigerated centrifuge, Yonglekang, Changsha, China) for 20 min at 4 °C, the precipitated crude synaptosomes were separated and resuspended in 3 mL of 0.32 mol/L sucrose (10 mmol/L Tris–HCl, pH 7.4). Finally, the aliquots of the freshly prepared synaptosomal suspension were used for treatment with fruit wines.

### 2.5. Treatment of Synaptosomes with Fruit Wines

First, the solutions of the fruit wines studied were prepared by dissolving 60 mg of the lyophilized samples in 600 μL of deionized water. Aliquots of the synaptosomal suspension (Section 2.1) were then incubated in the presence of the wine solutions at 37 °C for 10 min. Afterward, a freshly prepared H_2_O_2_ solution was added to reach a final concentration of 400 µmol/L [33] and the incubation was continued for 1 h. The treated synaptosomes were then used for subsequent monitoring of oxidative stress parameters—the activities of antioxidant enzymes (catalase, GPx, and SOD) and the extent of lipid peroxidation. An aliquot of synaptosomal suspension treated with H_2_O_2_ (without fruit wine) was used as a control.

### 2.6. Determination of Oxidative Stress Parameters

#### 2.6.1. Activities of Antioxidant Enzymes

Catalase activity was determined by following H_2_O_2_ consumption [34]. An amount of 2.975 mL of 50 mmol/L phosphate buffer (pH 7.0) containing 0.4 mmol/L EDTA-Na_2_ and 50 μL of synaptosomal sample were mixed in a quartz cuvette. The enzyme reaction was started by adding 30 μL of 3% H_2_O_2_ and the decrease in absorbance at 240 nm, which is proportional to H_2_O_2_ consumption, was observed for 5–10 min at room temperature. Specific catalase activity was expressed in U/mg protein (1 U = 1 µmol H_2_O_2_ consumed/min). Results were expressed as the mean of three repeated measurements of the same sample ± standard deviation.

For the determination of GPx activity, a coupled enzyme method based on monitoring NADPH consumption [32] was applied. First, a reaction mixture containing 8.9 mL of 50 mmol/L phosphate buffer (pH 7.0) with 0.1 mmol/L EDTA, 50 µL of 200 mmol/L GSH, 100 µL of glutathione reductase (100 units/mL), and 1 mg of NADPH was prepared. Then, 300 µL of synaptosomal sample was added to 3 mL of the reaction mixture and the enzyme reaction was started by adding 50 µL of freshly prepared 0.042% H_2_O_2_. The decrease in absorbance (λ = 340 nm), as a measure of NADPH consumption, was monitored for 5 min at room temperature. Specific GPx activity was expressed in U/mg protein. One GPx unit (U) catalyzes the H_2_O_2_-assisted oxidation of 1.0 mmol of reduced glutathione, GSH, to oxidized glutathione, GSSG, per 1 min. Results were expressed as the mean of three repeated measurements of the same sample ± standard deviation.

Total SOD activity (U/mg protein) was determined by SOD assay kit (code 19160) purchased from Sigma-Aldrich (Germany). Results were expressed as the mean value of three repeated measurements of the same sample ± standard deviation.

#### 2.6.2. Lipid Peroxidation

Lipid peroxidation was assessed by using the literature method [34] for measuring the reactive substance thiobarbituric acid (TBA) (equivalent of MDA) level as an indicator of membrane lipid damage. In brief, 0.5 mL of the synaptosomal sample was added to the mixture of 0.5 mL TBA (in 50 mmol/L NaOH) and 0.5 mL 25% HCl, and the glass tubes were heated in boiling water for 10 min. After cooling, 3 mL of *n*-butanol was added and centrifugation was carried out at 2000× *g* for 10 min. The separated organic phase (upper layer), containing the extracted chromogen, was further used for spectrophotometric measuring of absorbance (λ = 532 nm). 1,1,3,3-tetramethoxypropane was used as a standard. The extent of lipid peroxidation was expressed in nmol MDA/mg protein. Results were expressed as the mean value of three repeated measurements of the same sample ± standard deviation. The concentration of total proteins was quantified in the synaptosomal samples using the Lowry method [35]. Bovine serum albumin was used as a standard.

### 2.7. Briggs–Rauscher Reaction

The antioxidant activity of fruit wines was determined using the Briggs–Rauscher (BR) reaction [36]. The experiments were carried out in a stationary, well-stirring (σ = 900× *g*) reactor. The initial concentrations of the reactants were [CH_2_(COOH)_2_]_0_ = 0.0789 mol/L, [MnSO_4_]_0_ = 0.00752 mol/L, [HClO_4_]_0_ = 0.03 mol/L, [KIO_3_]_0_ = 0.0752 mol/L, and [H_2_O_2_]_0_ = 1.269 mol/L. The total volume of the BR reaction solution was 25 mL. The temperature of the reaction mixture was maintained at 37.2 °C using a flow-through thermostat. The reactants were added to the reaction vessel in the following order: malonic acid, manganese sulfate, perchloric acid, and potassium iodate. When the desired temperature of 37.2 °C was reached, H_2_O_2_ was added. Exactly 30 s after adding H_2_O_2_, i.e., after the start of the reaction, 50 µL of the wine sample was added. In this test, the fruit wines were analyzed without prior sample preparation, as ethanol showed no effect on the oscillation during the BR reaction [37]. The time evolution of the BR reaction over time was monitored potentiometrically using a Pt electrode as the measuring electrode and Hg/HgSO_4_ as the reference electrode. Results were presented as the mean of three repeated measurements of the same sample ± standard deviation.

### 2.8. Statistical Analysis

All statistical analyses were performed using R Statistical Software (version 4.4.1.; R Core Team 2024). Characterization data sets are presented as mean ± standard deviation. Descriptive statistics were calculated using three independent technical replicates. Statistical differences between groups were determined by three-way analysis of variance analysis (ANOVA) with a threshold of α = 0.05. Tukey post hoc tests were performed where applicable. The results of the descriptive statistics, ANOVA, and post hoc tests were determined using the R packages stats and multicompView. Principal component analysis (PCA) was used to analyze the variation and similarity of the compounds in the samples. Data adequacy for factor analysis and visualization was performed using some R packages (EFAtools, corrplot, ggcorrplot, ggplot2, ggfortify). Heatmap visualization and hierarchical cluster analysis were performed using the R package gplots to analyze the relationship between the fruit type and the measured associations.

## 3. Results and Discussion

### 3.1. Influence of Fruit Wines on the Activities of Superoxide Dismutase (SOD), Catalase (CAT), Glutathione Peroxidase (GPx), and Lipid Peroxidation in H_2_O_2_-Treated Synaptosomes

**Figure 1:**
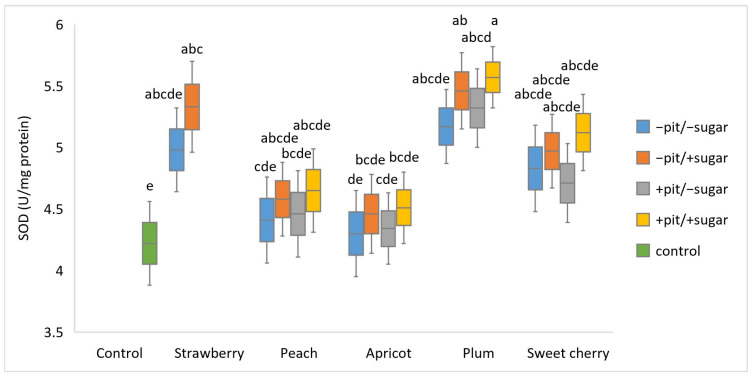
Boxplot of superoxide dismutase—SOD (U/mg protein) values (mean ± SD) measured in different types of fruit wines produced with or without pits (+pit; −pit) and with or without added sugar (+sugar; −sugar). a–e: Different lowercase letters indicate the significant difference between groups, which was confirmed by the ANOVA post hoc Tukey test (*p* < 0.05).

The determination of SOD activity was conducted in synaptosomes treated with lyophilized fruit wines, prepared by different technological procedures, and then experimentally induced oxidative stress with H_2_O_2_ (Figure 1). Of the SOD activities detected, the highest (5.57 U/mg protein) was observed in plum wine produced with the addition of sugar and pits (+sugar +pit), while apricot wine produced without the addition of sugar and pits (−sugar −pit) resulted in the lowest value (4.30 U/mg protein). Statistical analysis using three-way ANOVA showed a significant main effect of fruit (*p* < 0.001) and added sugar (*p* = 0.01), while no significant effect was detected for the presence of pit. The interactive effect in the three-way ANOVA was not statistically significant. It is important to emphasize that SOD activity was lower in the control (4.22 U/mg protein) compared to all fruit wines tested. Fruit wines produced with added sugar showed a better ability to increase SOD activity than the wines produced without added sugar. In specific samples such as plum wine produced with added sugar (+sugar) and with/without pits (+pit/−pit) and strawberry wine produced with added sugar (+sugar), a statistically significant difference was found compared to the control. The literature reported the pronounced SOD-like activities of extracts from three different plum varieties, which is in line with our results regarding the activity of plum wine [38]. It is also important to point out that feed enriched with strawberries was responsible for the increased activities of SOD, CAT, and GPx in the liver cells of rats. In the same study, the protective effects of strawberry extract against oxidative damage in human HepG2 cells were emphasized. The activities of antioxidant enzymes (SOD, GPx, and CAT) were increased compared to the cells that were not treated with extracts [14].

**Figure 2:**
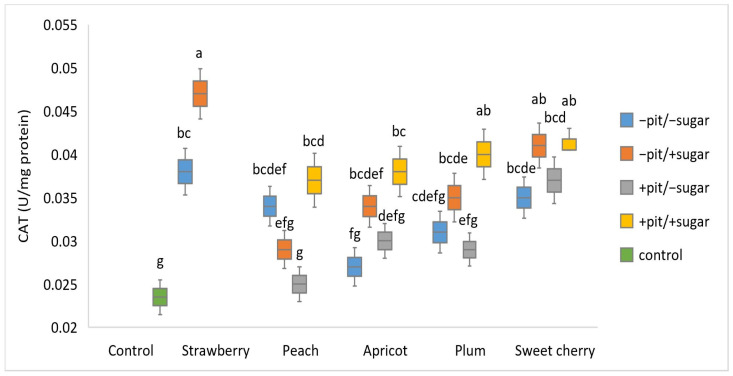
Boxplot of catalase—CAT (U/mg protein) values (mean ± SD) measured in different types of fruit wines produced with or without pit (+pit; −pit) and with or without added sugar (+sugar; −sugar). a–g: Different lowercase letters indicate the significant difference between the groups, confirmed by the ANOVA post hoc Tukey test (*p* < 0.05).

The determination of CAT activity was conducted in synaptosomes treated with lyophilized fruit wines prepared with a different technological approach, in which oxidative stress was experimentally induced with H_2_O_2_ (Figure 2). Compared to the corresponding control (0.024 U/mg protein), synaptosomes treated with all fruit wines increased CAT activity. The highest CAT activity (0.047 U/mg protein) was observed in synaptosomes treated with strawberry wine prepared with added sugar (+sugar), while conversely, the lowest activity (0.025 U/mg protein) was observed in peach wine prepared with added sugar without pits (+sugar −pit). After the statistical analysis of CAT values by three-way ANOVA, a significant interactive effect of the factors was found (*p* < 0.001). A significant main effect was found for each factor: type of fruit (*p* < 0.001), added sugar (*p* < 0.001), and presence of a pit (*p* = 0.05). For all wines analyzed, except peach wine, higher CAT activities were observed in samples with added sugar. It is interesting to emphasize that a statistically significant difference from the control was observed in all strawberry and sweet cherry wines, while in apricot and plum, it was only observed in the samples with added sugar. In agreement with our results, another study showed that oxidative stress parameters such as the activities of antioxidant enzymes CAT, GPx, and SOD were increased in the group of participants who consumed strawberry powder obtained after freeze-drying. The presence of strawberries in the human diet significantly improved the antioxidant capacity of serum and its scavenging activity against ROS [39]. Our previous findings [31] regarding the phenolic profile of peach wine are consistent with the literature data that hydroxycinnamic acids (chlorogenic acid) and flavonoids (epicatechin and quercetin) are the predominant phenolic compounds in peach [40,41]. The ability of peach fruit and derived products such as wine to prevent oxidative stress and activate antioxidant protective enzymes is highly dependent on their phenolic profile. In contrast to our results, another study reported that in synaptosomes exposed to oxidative stress, the activities of CAT and SOD were decreased after treatment with the phenolic fraction of *Croton zambesicus* leaves [42].

**Figure 3:**
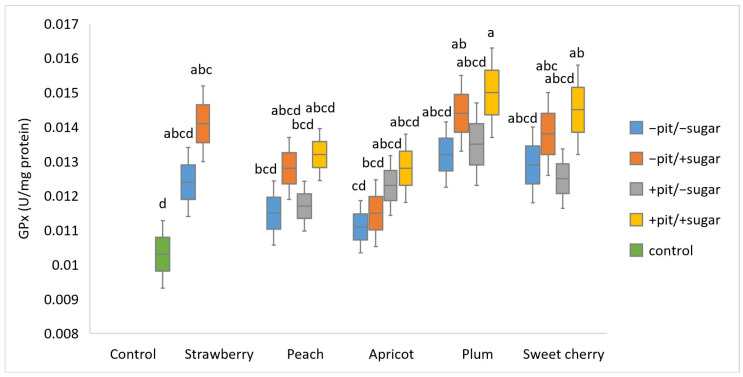
Boxplot of glutathione peroxidase—GPx values (U/mg protein) (mean ± SD) measured in different types of fruit wines produced with or without pits (+pit; −pit) and with or without added sugar (+sugar; −sugar). a–d: Different lowercase letters indicate the significant difference between groups, confirmed by the ANOVA post hoc Tukey test (*p* < 0.05).

The determination of GPx activity was conducted in synaptosomes treated with lyophilized fruit wines produced by a different technological approach, in which oxidative stress was experimentally induced with H_2_O_2_ (Figure 3). Synaptosomes treated with all fruit wines showed higher values of GPx activity compared to the corresponding control (0.0103 U/mg protein). The highest GPx activity (0.015 U/mg protein) was observed in plum wine with added sugar and pits (+sugar +pit), while it was lowest in apricot wine without sugar and pits (−sugar −pit) (0.0111 U/mg protein). The three-way ANOVA showed the significant main effects of fruit type (*p* < 0.001) and added sugar (*p* < 0.001) on the GPx activity observed after the treatment of the synaptosomes with different wines.

A significant interaction effect of these two factors was not observed. Among the wines analyzed, it should be emphasized that strawberry, plum, and sweet cherry wines produced with added sugar showed a significant difference compared to the control. The ability of plum wine to increase GPx activity is due to the presence of biologically active compounds, among which polyphenols stand out. The results of our previous study point to chlorogenic acid as the predominant phenolic compound in plum wine [31]. This result is consistent with the literature data indicating a high chlorogenic acid content in plums. In addition, chlorogenic acid has been reported to have antioxidant properties and can prevent oxidative stress as a ROS scavenger. Chlorogenic acid was found to be responsible for the modulation of GPx, CAT, and SOD activities, which had an effect on the suppression of oxidative stress in serum and mucosal samples from rats [43]. In contrast to our results, a study conducted in different cell models showed that plum extracts decreased the activities of GPx, SOD, and CAT in mice with benzo(a)pyrene-induced oxidative stress [16]. The studied strawberry wine also showed a good ability to increase GPx activity due to its high phenolic content [31]. As biologically active compounds, polyphenols play an important role as modulators of antioxidant enzyme activities. This fact is confirmed by the study that revealed higher activities of GPx, CAT, and SOD in rat cells after a strawberry-enriched diet [10,14]. Apart from the fact that apricot wine exhibited the weakest effect on increasing GPx activity, the literature data indicate a strong protective effect of apricots on induced oxidative damage in the rat small intestine. The apricot-fed rat group, in which intestinal injury was induced by methotrexate, showed significantly increased activities of GPx, CAT, and SOD compared to the group treated with methotrexate alone [44].

**Figure 4:**
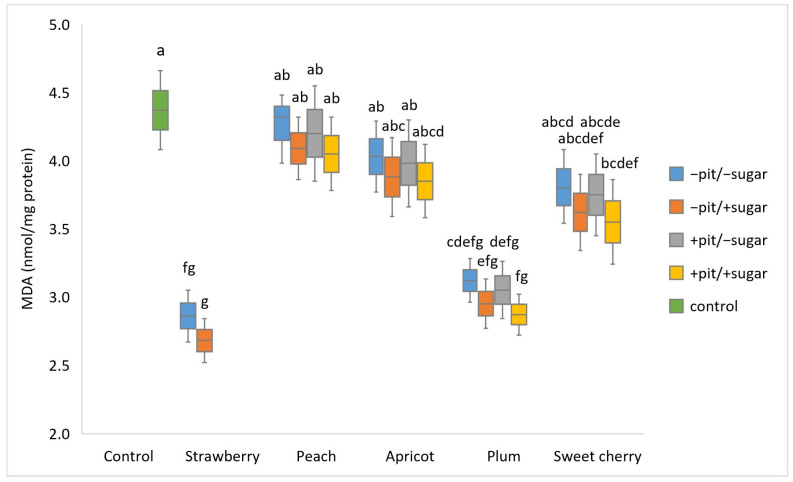
Boxplot of malondialdehyde—MDA values (nmol/mg protein) (mean ± SD) measured in different types of fruit wines produced with or without pits (+pit; −pit) and with or without added sugar (+sugar; −sugar). a–g: Different lowercase letters indicate the significant difference between groups confirmed by the ANOVA post hoc Tukey test (*p* < 0.05).

In the synaptosomes exposed to the lyophilized fruit wines and in which oxidative stress was subsequently induced, the MDA content was detected, which is an indicator of membrane damage and lipid peroxidation (Figure 4). The MDA values in the synaptosomes treated with all fruit wines were lower compared to the corresponding control (4.37 nmol/mg). Strawberry wine produced with added sugar (+sugar) showed the best ability to lower MDA level (2.68 nmol/mg), while the highest MDA content (4.26 nmol/mg) was observed in synaptosomes treated with peach wine produced without added sugar and pit (−sugar −pit). The three-way ANOVA used for statistical analysis showed significant main effects of fruit type (*p* < 0.001) and added sugar (*p* = 0.02), while for pits, it was not observed. The interaction effect for these three factors was not significant. Wine produced with added sugar showed a better ability to lower MDA content. Interestingly, the MDA levels of strawberry, plum, and sweet cherry wine (+sugar +pit) were statistically significantly different from those of the control. In line with our results, another study showed that strawberry consumption lowered MDA levels in human serum [39]. The results of this study regarding the activity of the analyzed plum wine in lowering MDA levels are consistent with the literature data emphasizing the ability of plum extracts to lower lipid peroxidation levels [38]. The health-promoting effect of another plum product, plum juice, was investigated in a crossover study and was shown to lower MDA levels and increase the antioxidant capacity of urine [45,46].

Moreover, the literature data indicate a reduction in MDA values in the fraction of oxidized LDL particles treated with plum and peach juice, supporting our findings on the ability of wines produced from these two fruits to reduce MDA levels during oxidative stress. As a source of polyphenols, plum and peach juice prevented inflammatory processes and oxidative stress in the liver and heart by inhibiting lipid and triglyceride synthesis [47]. The outstanding biological activity of sweet cherry wine from this study is consistent with data from the literature. The study conducted on rats showed that the diet supplemented with sweet cherry significantly reduced MDA levels and ROS in the liver of the experimental animals compared to the control group. This activity is attributed to the nutritional composition of sweet cherry, which is a rich source of biologically active compounds such as polyphenols, vitamins, fibers, and minerals [48]. As a beverage that does not undergo thermal treatment during the production process, sweet cherry wine is certainly a rich source of the above compounds. The ability of peach to lower MDA levels found in our study is consistent with the literature data showing that oxidative stress induced in rat kidney tissue by CCl_4_ is responsible for higher MDA content, which was lowered after the administration of peach even at low doses. This finding offers the possibility of considering peaches and peach-derived products such as wine as a potential solution to prevent oxidative stress-induced kidney problems [41]. In addition to the fact that apricot wines showed a lower ability to lower MDA, our findings are supported by the literature data, indicating that apricot fruit has the ability to decrease MDA levels in various models of rat tissues [44,49].

The biological activity of lyophilized fruit wines in the prevention of oxidative stress and lipid peroxidation results from the active principles, among which polyphenols stand out. In addition to wines, the phenolic content of lyophilization products obtained is almost identical to that of the wines, and thus these types of samples are suitable for the estimation of biological activity in vitro. The polyphenols from the fruit wines contributed to the obtained increased activity of antioxidant enzymes and to the reduction of MDA values in the synaptosomes treated with the corresponding lyophilized fruit wines. Our previous study [31] analyzed the phenolic profile of fruit wines used to treat synaptosomes subjected to experimentally induced oxidative stress. Among the polyphenols, the highest content of phenolic acids (chlorogenic acid, gallic acid, protocatechuic acid, and *p*-hydroxybenzoic acid) and flavonoids (catechin, epicatechin, and quercetin) was found, while ellagic acid was quantified only in strawberry wine. Wines made from fruits with dark skin (plum, sweet cherry, and strawberry) showed a higher content of phenolic compounds. The addition of sugar before the start of fermentation increased ethanol content, which improved the extraction of phenolic compounds from skins and seeds during fermentation.

In agreement with our results, a high content of chlorogenic acid was found in stone fruits. The plum extract showed a strong activity as an inhibitor of hydrogen peroxide and superoxide radicals due to the presence of many biologically active compounds, of which chlorogenic acid can be emphasized as the most dominant. The same extract had a significant content of rutin and caffeic acid, which were also present in our samples of plum wine [50]. The capacity of rutin to prevent oxidative stress damage was demonstrated in a rat nerve tissue model. After exposure to fluoride, reduced activities of SOD, CAT, and GPx were observed in the cerebrum and brain layer of rats. In the same brain areas, rutin significantly increased the activities of the aforementioned antioxidant enzymes compared to the control. Fluoride treatment, which increased MDA levels in the cerebrum and brain layer of rats, was decreased in the presence of rutin [51]. Furthermore, in an animal model in which a neurodegenerative process was induced by 3-nitropropionic acid, it was shown that treatment with rutin increased the activities of SOD, CAT, and GPx in the striatum, while the MDA level was decreased [52]. Similarly, rutin and caffeic acid showed the ability to decrease the MDA level and increase the activity of SOD in neurons [53]. These results suggest that both rutin and caffeic acid could be used to prevent the development of neurodegenerative diseases. The pretreatment of HepG2 cells with the phenolic fraction from olive leaves significantly reduced H_2_O_2_ -induced oxidative stress and protected against cell injury. Moreover, the same extract showed the ability to increase the activities of antioxidant protection enzymes (SOD, CAT, and GPx) and decrease the MDA amount [54].

Our earlier results [31] are consistent with the literature reporting the phenolic acid content of sweet cherries. It is emphasized that among the hydroxycinnamic acids, chlorogenic acid was detected in the highest amount, while the content of hydroxybenzoic acid was lower, and the most significant compound from this group was gallic acid. The predominant flavonoids of sweet cherry were catechin, epicatechin, and quercetin [55], which is consistent with our results. The previously mentioned phenolic compounds contribute to the potential of sweet cherry to prevent oxidative stress in synaptosomes. The antioxidant activity of these compounds against oxidative stress has been demonstrated in various cell models. In experimentally induced oxidative stress in rat cells, quercetin treatment increased SOD and CAT activities, while the MDA level was decreased [56,57]. Phenolic compounds from sweet cherry showed a potent activity in preventing oxidative stress. The pretreatment of HepG2 cells with sweet cherry extracts, which are rich in phenolic compounds, protected against oxidative damage [58]. In another study, it was reported that H_2_O_2_-induced oxidative damage of rat HepG2 cells was reduced in the presence of catechin and quercetin. These two polyphenols increased the activities of SOD, CAT, and GPx, while the MDA level was significantly decreased. These two polyphenols increased the activities of SOD, CAT, and GPx, while the level of MDA was remarkably decreased [11]. The presence of epicatechin prevented oxidative stress in the liver tissue of mice exposed to the toxin. Increased activities of antioxidant enzymes (SOD, CAT, and GPx) and a decrease in MDA levels were observed in epicatechin-treated cells compared to cells in the control group [59]. Other than the fruits, other plants such as *Hemerocallis fulva* also showed a significant protective effect against oxidative damage. As a rich source of flavonoids, the leaf extracts of this plant reduced ROS levels and showed satisfactory antioxidant activity [60].

The ability of strawberry wine to increase the activities of SOD and GPx may be due to ellagic acid, which is the most abundant compound in this wine [9,61]. The biological activity of ellagic acid has been demonstrated both in vitro and in vivo, including its ability to increase CAT activity and decrease MDA levels [62]. Strawberry wine was found to contain the highest level of chlorogenic and gallic acid, as well as rutin. Various experimental models have shown the protective effect of gallic acid against oxidative stress. After oxidative damage induced by streptozotocin and AlCl_3_, the administration of gallic acid increased the activities of SOD, CAT, and GPx and decreased MDA levels [63,64]. Furthermore, it is important to emphasize the positive effect of chlorogenic acid on the cardiovascular system, as it was the most abundant compound in all fruit wines from this study. Compared to the control group, rats that received chlorogenic acid before myocardial injury showed significantly increased SOD and CAT activities in plasma, reducing oxidative stress [12]. The phenolic extracts obtained from various plant organs (fruits, leaves, and flower buds) of *Vaccinium dunalianum* showed neuroprotective properties against hydrogen peroxide-induced damage in PC12 cells, while free radical formation was reduced. Chlorogenic acid, as the predominant compound in these extracts, contributed significantly to the protective effects against oxidative stress [65].

The beneficial health effects of peach and apricot are partly due to the phenolic compounds responsible for the antioxidant properties of these fruits and their products. Particularly noteworthy are the phenolic acids (chlorogenic and protocatechuic acid) and the flavonoids (quercetin and rutin), which contribute significantly to this effect [66,67]. These compounds, which were also present in the peach and apricot wine samples studied, contributed to the ability of these wines to increase the activity of antioxidant protection enzymes. The presence of phenolic acids in various natural products is responsible for the increased activity of antioxidant protective enzymes (SOD, CAT, and GPx) and the ability of intracellular antioxidants (as free radical scavengers) to protect against free radicals [68]. The protective effect of protocatechuic acid against oxidative stress induced by H_2_O_2_ in PC12 cells was due to an increase in CAT, GPx, and SOD activities and a decrease in MDA levels, which prevented the cytotoxic effect and increased cell viability [69,70]. In contrast to our results, another study reported that lyophilized peach samples decreased the activities of CAT and SOD in rat liver and kidney tissues during induced oxidative stress after treatment with CCl_4_. This effect could be explained by the fact that phenolic and other antioxidant compounds from peach samples were responsible for free radical scavenging in different tissue model systems, which did not result in the increased activity of these enzymes [41].

### 3.2. PCA Analysis—Interrelationship Between Antioxidant Properties and Oxidative Stress Parameters

**Figure 5:**
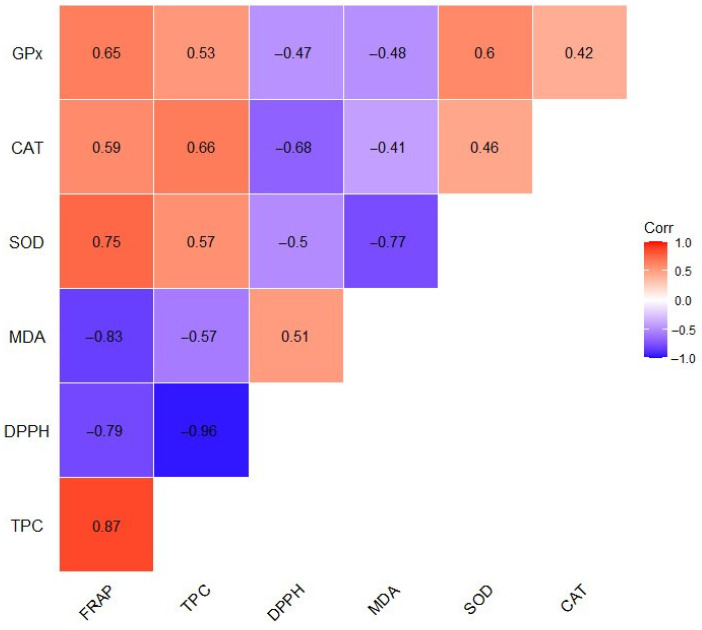
Graphical representation of the correlation heat map between the oxidative stress parameters (SOD, CAT, GPx, and MDA) and the antioxidant properties (FRAP, TPC, and DPPH). Red indicates positive and blue represents negative correlation coefficients. All correlation coefficients are statistically significant at the level of 0.05. SOD—superoxide dismutase, CAT—catalase, GPx—glutathione peroxidase, MDA—malondialdehyde, FRAP—ferric reducing ability of plasma, TPC—total phenolic content, DPPH—2,2-diphenyl-1-picrylhydrazyl.

Principal component analysis (PCA) was conducted to obtain an overview of the interrelationship between oxidative stress parameters (CAT, GPx, SOD, and MDA) (Figure 1, Figure 2, Figure 3 and Figure 4) and antioxidant activity (FRAP), total phenolic content (TPC), and antiradical activity (DPPH) from our prior study [31]. The adequacy of the data for factor analysis was tested using the Kaiser–Meyer–Olkin test (KMO = 0.75) and Bartlett’s test for sphericity (*p* < 0.001). All correlation values between the observed compounds were statistically significant (*p* < 0.05) and are shown in Figure 5. Red indicates a positive correlation, while blue indicates a negative correlation.

**Figure 6:**
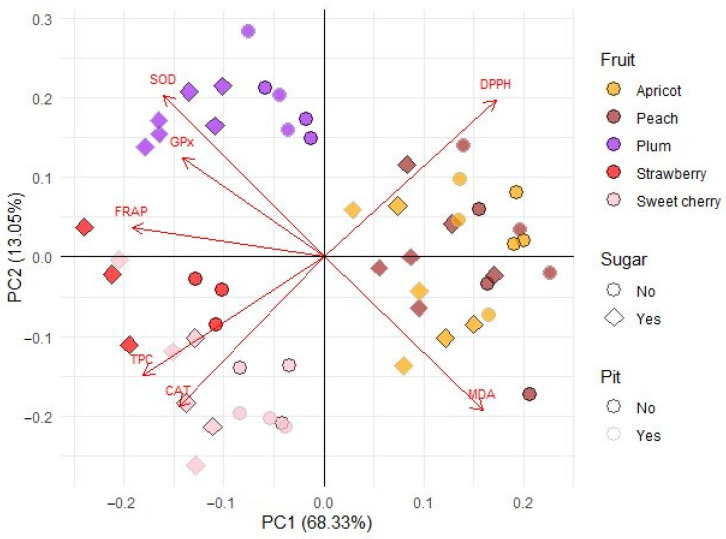
Biplot from the principal component analysis. The arrows represent the loadings of the variables they represent, and the dots represent the values of the principal components for the different types of fruit wines shown along the first two components. The types of fruit wine are represented by different colors. Square and circular shapes represent the presence or absence of sugar. Light and dark edges of the dots represent the presence or absence of pits. SOD—superoxide dismutase, CAT—catalase, GPx—glutathione peroxidase, MDA—malondialdehyde, FRAP—ferric reducing ability of plasma, TPC—total phenolic content, DPPH—2,2-diphenyl-1-picrylhydrazyl.

After performing PCA, there were two components that individually explained more than 10% of the variance and cumulatively 81.38% (Comp 1 and Comp 2) of the diversity (Figure 6). To maximize the availability of these two components to explain the existing variables, a varimax rotation was performed. After rotation, it was found that the variables TPC and CAT showed similar dynamics, but were opposite in comparison to DPPH.

The values of TPC and CAT for strawberry and sweet cherry wines were the highest among the other fruit wines, while the two aforementioned wines showed the best anti-radical activity against DPPH radicals. In contrast, the MDA variable showed an opposite behavior compared to the values of SOD, GPx, and FRAP. It is important to note that apricot and peach wines were grouped together, as these wines showed the lowest ability to reduce MDA and the lowest anti-radical activity against DPPH radicals. Plum wines were grouped separately, as they showed the highest increase in SOD and GPx in this study.

### 3.3. HCA Analysis—Interrelationship Between Antioxidant Properties and Oxidative Stress Parameters

**Figure 7:**
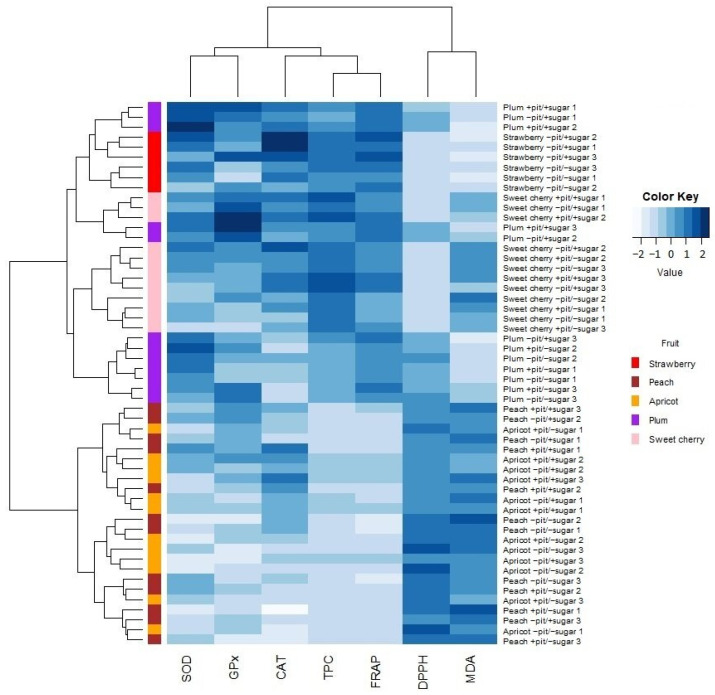
Hierarchical cluster analysis with the correlation heat map showing the relationship between oxidative stress parameters and antioxidant properties (x-axis) and the different types of fruit wines produced with or without pits and with or without added sugar (y-axis). SOD—superoxide dismutase, CAT—catalase, GPx—glutathione peroxidase, MDA—malondialdehyde, FRAP—ferric reducing ability of plasma, TPC—total phenolic content, DPPH—2,2-diphenyl-1-picrylhydrazyl.

The hierarchical cluster analysis (HCA) was conducted with the same variables as those in the PCA analysis. The heat map shows the hierarchical clustering of the numerical data subjected to different measurements (see Figure 7). Given the different scales of parameter measurement, all data were standardized before displaying. Thus, the observation scale ranges from −2 to 2, with the white color representing lower values and the dark blue color representing higher registered values. According to the clustering results, the observed fruit wines were divided into two main categories. The first group included wines made from strawberry, plum, and sweet cherry. Upon examining this first group, subcategories were identified based on the presence or absence of sugar before the start of fermentation. This result confirms that the addition of sugar influences the behavior of the analyzed parameters. The second group included wines made from apricot and peach, which were also divided into subcategories depending on whether sugar was present or not. As already mentioned, strawberry, plum, and sweet cherry were organized in the first group, which had a darker skin color compared to peach and apricot. The wines of the first group showed better results in oxidative stress parameters and antioxidant properties than those from the second group.

### 3.4. Effect of Fruit Wines on the Briggs–Rauscher Reaction Dynamics

**Figure 8:**
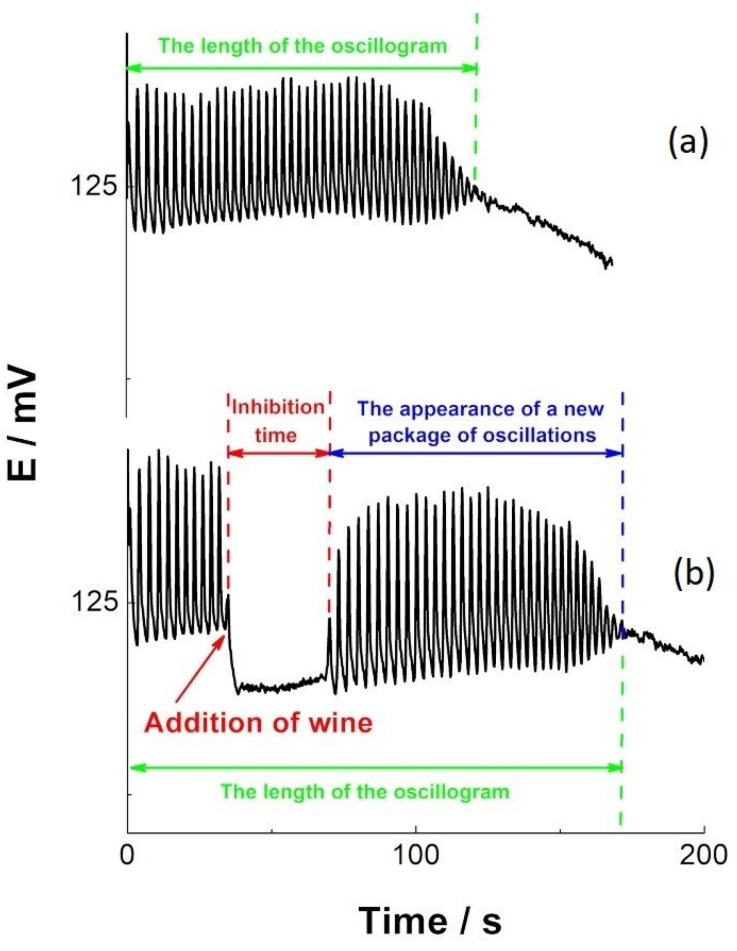
The Briggs–Rauscher reaction was followed potentiometrically; (**a**) the basic BR oscillogram obtained without the addition of wine; (**b**) the BR dynamics caused by the addition of wine (inhibition time and the appearance of new oscillation packets).

The Briggs–Rauscher (BR) oscillogram without added wine (Figure 8a) showed oscillatory dynamics that started immediately after adding hydrogen peroxide and lasted for 121 ± 10 s. All analyzed fruit wine samples influenced the BR oscillatory dynamics in the same way (Figure 8b). As shown in Figure 8b, the addition of wine (30 s after initiating the oscillatory reaction) immediately stopped the oscillatory behavior of the BR. After this period, called the inhibition time, the response system can develop oscillatory behavior again, and in this case, a new set of oscillations has emerged (Figure 8b).

**Figure 9:**
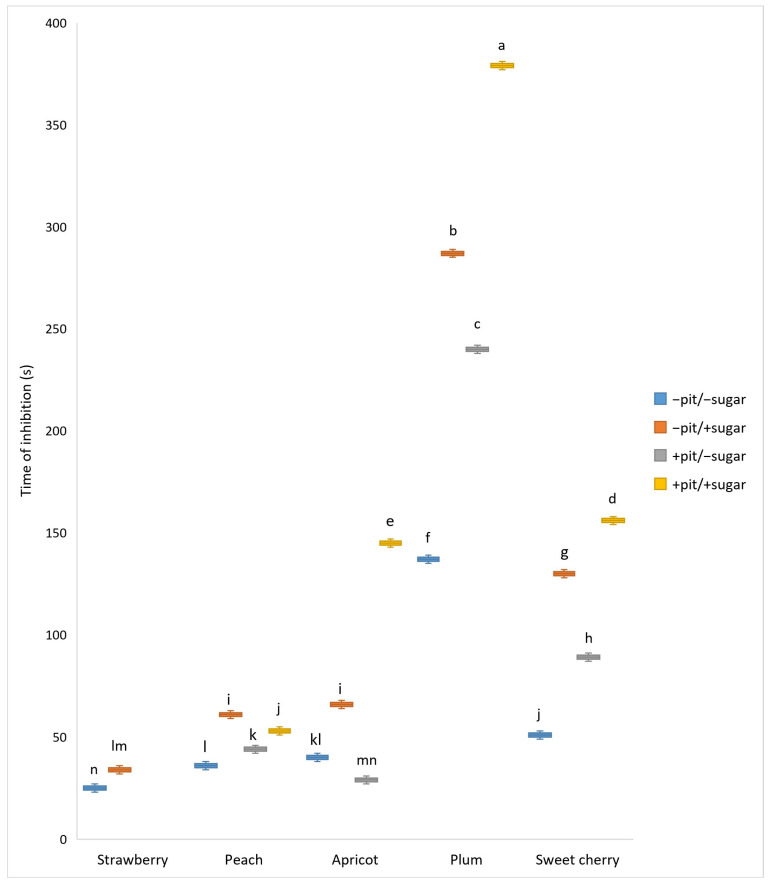
Boxplot of inhibition time (s) values (mean ± SD) measured for different types of fruit wines produced with or without pits and with or without added sugar. a–n: Different lowercase letters indicate the significant difference between the groups, which was confirmed by ANOVA post hoc Tukey test (*p* < 0.05).

The addition of all selected wines stopped the oscillating behavior. The results of the following BR reaction are shown in Figure 9. However, the best inhibitory activity (397 s) was shown for plum wine produced with the addition of sugar and pits (+sugar +pit), while the lowest (25 s) was observed for strawberry wine without added sugar (−sugar). The three-way ANOVA revealed a statistically significant interaction effect of the factors (*p* < 0.001), as well as the significant main effects of each factor (fruit, sugar, and pit) separately (*p* < 0.001 for each factor). The minimal effect of fruit type on the inhibitory activity was observed for strawberry wine, while the effect was greatest for plum wine. Wines produced with added sugar showed better inhibitory activity than those produced without added sugar. The presence of pits significantly increased the inhibitory activity of the fruit wines. This finding could be explained by the results of our prior study [31], which showed that wines produced with added sugar and pits had higher levels of specific phenolic compounds and better antioxidant activity. The addition of sugar before the start of fermentation contributed to a higher ethanol content in the wine, which enhanced the extraction of phenolic compounds from the solid parts of the fruit (skin, pit, and seeds). The different values for the inhibitory effects can be attributed to the different phenolic profiles of the fruit wines, in which phenolic acids and flavonoids were predominant [31]. The following literature data indicate that both the phenolic acids (hydroxycinnamic acid and hydroxybenzoic acid derivatives) and the flavonoids have a significant effect on the suppression of BR oscillations.

In agreement with this, the literature results indicated high inhibitory activity for chlorogenic and caffeic acids, while a lower activity was observed for gallic and ellagic acids. Among the flavonoids, rutin showed higher activity compared to quercetin [71]. In another study, the highest inhibitory activity in the BR reaction was found for chlorogenic acid [22], while the activity was observed for caffeic acid in the same reaction [72]. All these phenolic compounds were present in the fruit wines studied and contributed significantly to the scavenging properties observed using the BR method. Our results emphasize the suitability of the BR method for evaluation of the antioxidant activity of fruit wines, which is based on the synergistic effect of all biologically active compounds.

The in vitro experiments performed in this study have shown that fruit wines are able to reduce cell damage caused by oxidative stress. The limitations of this study include the lack of evaluation of the bioavailability and metabolism of phenolic and other biologically active compounds responsible for the antioxidant effect of fruit wines. Accordingly, appropriate in vivo studies are needed to gain a broader perspective on the ability of these biologically active compounds to protect against free radicals.

## 4. Conclusions

The results of this study demonstrate, for the first time, the protective effects of strawberry and drupe fruit wines against H_2_O_2_-induced oxidative stress in rat synaptosomes. In addition, the BR method revealed, for the first time, the antioxidant properties of wines produced from these fruits. Strawberry and plum wines exhibited the highest activity of antioxidant enzymes and showed the strongest ability to reduce damage to the synaptosomal lipid membrane, while the highest inhibitory activity was observed in plum and sweet cherry wines using the BR method. The ROS-scavenging activity of fruit wines results from the synergistic effect of various natural active compounds that possess antioxidant properties and are able to prevent oxidative stress-induced diseases. This study provides a valuable perspective into the in vitro protective effects of fruit wines against oxidative stress and provides an essential basis for further in vivo studies.

## Data Availability

The data presented in this study are available on request from the corresponding author.
